# Coronary CT angiography in acute chest pain

**DOI:** 10.12688/f1000research.11250.1

**Published:** 2017-07-13

**Authors:** Nikhil Goyal, Arthur Stillman

**Affiliations:** 1 Department of Radiology and Imaging Sciences, Emory University, Atlanta, GA, USA

**Keywords:** coronary computed tomographic angiography, acute chest pain, ischemic disease, acute coronary syndrome

## Abstract

Coronary computed tomographic angiography has become a reliable diagnostic tool in the evaluation of patients with chest pain. Studies have shown this modality to be accurate and safe when compared with conventional methods of assessing patients with chest pain. We review the recent developments with coronary computed tomographic angiography and devote particular attention toward its application to triage patients in the emergency department.

## Introduction

Coronary computed tomographic angiography (CCTA) has emerged in the last 15 years as a non-invasive method to evaluate the coronary arteries, cardiac chambers, and valves. The use of electrocardiography gating permits synchronization of data acquisition to the cardiac cycle to minimize motion of the coronary arteries and cardiac structures.

Recent emphasis has been on the application of CCTA in the triage of low-risk chest pain patients presenting acutely to the emergency room. Randomized trials have shown that coronary angiography in the emergency department (ED) setting reduces length of stay, initial cost in the ED, and time to discharge when compared with the current standard of care with equivalent safety and accuracy. However, there are data showing that the initial cost savings may be offset by additional downstream testing and sometimes overall higher radiation dose when CCTA is used. Despite this, the potential use of CCTA in the ED setting is promising, especially given some of the newest developments that may curb the need for additional testing.

With advancements such as increasing number of detectors and x-ray tubes as well as advances in software (perfusion imaging and computed tomographic–fractional flow reserve, or CT-FFR), CCTA may offer the opportunity of having a “one-stop shop” for evaluating select patient populations.

## Background and evolution of coronary computed tomographic angiography

Current CT scanners used for coronary angiography have superior spatial and temporal resolution when compared with older-model scanners with acceptable radiation exposure. With detector ranges of 64 to 320 detectors and a detector width of 0.5 to 0.7 mm, current scanners can provide wide coverage and thin slice acquisition. With the advent of dual-source scanners and the use of multi-segment reconstruction algorithms, temporal resolution has also improved, thus allowing scanning of faster and (if necessary) irregular heart rates. However, it should be stated that the use of beta-blockers remains common practice and should be used when necessary to obtain optimal images at the lowest possible radiation dose. The accuracy of CCTA when compared with conventional angiography has been well established in multi-center trials as well as in meta-analysis studies. The high negative predictive value (NPV) of 83–99% ^1– 3^ and the corresponding negative likelihood ratio of 0.02 ^4^ most importantly can rule out obstructive coronary artery disease or myocardial ischemia. The positive predictive value (PPV) of CCTA to detect myocardial ischemia is less ^5^, and about 50% of cases with plaque causing more than 50% narrowing have an abnormal result on myocardial perfusion imaging or demonstrate ischemia by FFR. Thus, the strength of this modality currently lies in its ability to rule out ischemic disease rather than rule it in.

In addition to investigating its diagnostic accuracy, several studies have investigated the prognostic efficacy of CCTA. Computed tomographic angiography (CTA) is a good predictor of risk, and its most notable feature is predicting excellent outcomes for people with normal results. Long-term data now exist for excellent prognosis beyond 5 years for patients with normal CTA results with a negative likelihood ratio of 0.008 for major adverse cardiac event (MACE) for a normal CTA result ^6– 9^. Not surprisingly, risk for MACE increases incrementally as plaque burden increases ^6– 9^ and the more proximal a plaque is within the coronary tree, the worse the prognosis. ^5, 10, 11^. This has been documented up to 7 years post-scan ^12^. Overall, these findings are not unexpected and reflect what has already been known from conventional angiography.

## The chest pain problem

While the role of CTA in the outpatient setting has been established, much of the recent work has centered on the use of CTA in patients presenting to the ED with acute chest pain. The problem of acute chest pain is a major source of resource utilization in the ED. It is estimated that over 8 million visits to the emergency room are for chest pain ^13^. The current model of admitting patients to the hospital to “rule out acute coronary syndrome (ACS)” with serial biomarkers and functional assessment is both time- and cost-inefficient and leads to overcrowding in hospitals. The majority of patients who are admitted under these circumstances do not have ACS. Additionally, a small fraction of patients are incorrectly discharged ^13^ with a corresponding increase in mortality ^14^. Given that the strength of CCTA is its high NPV, several investigators have applied the use of CCTA to low- to intermediate-risk chest pain patients presenting to the emergency room.

To date, three large clinical trials have demonstrated that CCTA is a safe and effective means to discharge low-risk patients from the ED ^15– 17^. The CT-STAT (Coronary Computed Tomographic Angiography for Systematic Triage of Acute Chest Pain Patients to Treatment) trial demonstrated a 54% reduction in time to diagnosis and a 38% reduction in ED costs between CCTA and myocardial perfusion imaging with no difference in a safety endpoint of MACE at 60 days ^15^. Litt *et al*., in the ACRIN-PA trial ^16^, demonstrated a 50% increase in discharge home and no increase in MACE at 30 days in patients with a normal CCTA when comparing CCTA with standard of care. Finally, the ROMICAT II (Rule Out Myocardial Infarction/Ischemia Using Computer Assisted Tomography II) trial demonstrated decreased length of stay (8 versus 26 hours), fourfold increase in rates of direct discharge from the ED, and no difference in MACE at 28 days ^17^. However, it should be noted that cumulative costs at 28 days were higher in the CCTA group, as more patients in the CTA group underwent additional tests after initial discharge. A meta-analysis by Hulten *et al*. demonstrated that the use of CCTA in the ED led to an overall increase in downstream use of conventional angiography and revascularization by 2% when compared with usual care ^18^. This is likely because of the high sensitivity of CTA in finding obstructive disease that required further testing to clarify whether these lesions were flow-limiting. In patients with subclinical atherosclerosis identified on CCTA in the ED, there may be potential benefit in the form of increased use of preventive measures, although initiating these measures remains challenging in the ED setting.

Longer-term data now exist for the “warranty” period of a “normal” CCTA reading for patients presenting to the ED. Hollander *et al*. showed that, at 1 year following a CTA in the ED, none of the 481 patients with less than 50% stenosis and an ejection fraction of more than 30% had a non-fatal myocardial infarction or needed revascularization ^19^. Schlett *et al*. published 2-year data for patients from the original ROMICAT trial and showed that patients with a normal ED CTA had a 0% 2-year risk for MACE ^20^. It is important to recognize that patients with non-obstructive coronary disease in this study had a 4% 2-year risk for MACE risk (which is non-trivial and requires appropriate therapy).

Additional data also show that there are reduced return visits to the ED in patients who undergo CCTA ^21^. In the 2010 appropriate use criteria, CCTA is considered an appropriate indication for acute chest pain in low- to intermediate-risk patients.

It is fairly evident that patients who have normal (or near normal) CTA results can be safely discharged from the ED as not having ACS. CCTA has an NPV of nearly 100% in low-risk patients in this patient group ^22, 23^. Meta-analysis demonstrates an associated negative likelihood ratio of 0.06 ^24^. Most physicians also agree that patients with obstructive disease should go on for further testing. However, it remains unclear what to do with patients who exhibit intermediate lesions that *may* be flow-limiting. A lot of recent focus has centered on obtaining functional data as part of the CT exam in order to further assess these “intermediate” lesions. After all, combined anatomical assessment of the coronaries and their corresponding effect on perfusion remain the most comprehensive and desirable evaluation of the heart. The two forms of investigation recently developed to address this issue are CT-FFR and CT perfusion scanning.

In the catheterization lab, intermediate lesions are assessed by passing a pressure transducer wire across the lesion and recording pressures distal and proximal to the lesion in question. Adenosine is administered to induce hyperemia. This process is called measuring FFR. This is important because FFR measurement has been shown to better guide percutaneous coronary intervention therapy with more favorable outcomes than angiographic appearance alone ^25^. For reference, an FFR value below 0.80 is considered flow-limiting.

Given the invasive nature of conventional FFR, investigators sought out a non-invasive equivalent ^26– 28^. The physics of simulating invasive pressure measurements is complex and involves the application of computational fluid dynamics equations to blood flowing within the coronary vessels. CT output data can be transmitted to supercomputers which can perform the necessary mathematics and generate a CT-FFR measurement. Two large trials have investigated the effect of computing FFR measurements from CTA data on sensitivity, specificity, PPV, and NPV profiles of CTA while using invasive FFR as the gold-standard comparison. The DISCOVER-FLOW (Diagnosis of Ischemia-Causing Stenoses Obtained Via Noninvasive Fractional Flow Reserve) trial ^29^ demonstrated improvements in specificity (25→82%) and PPV (58→85%). The NXT trial ^30^ showed improvements in specificity and PPV (32→84% and 33→67%, respectively). Since then, several vendor- or institutional-specific FFR computational programs have been devised and some have been tested in single-center trials with similar results. Overall, the data from the multi-center and single-center local software trials demonstrate improvements in the area under the receiver-operator characteristic curves when evaluating CT-FFR versus CTA alone compared with invasive FFR. However, it should be noted that the computational power currently required to generate CT-FFR results necessitates a long (for example, up to 24 hours by some vendors) calculation time and thus is not suitable for use in EDs. Further work may reveal faster computation algorithms, making this modality more useful in the ED setting.

Hemodynamic evaluation via assessment of myocardial perfusion is also a rapidly developing area of study. CT perfusion (CTP), like a provocative nuclear test, involves both a rest and a stress phase, and several vasodilator agents, including dipyridamole, adenosine, and regadenoson, can be used. Myocardial perfusion is determined by visual analysis assessing for areas of myocardial hypoattenuation, although several semi-quantitative techniques are now available. Numerous studies have shown the efficacy of perfusion CT alone with sensitivity ranges from 50% to 96%, specificity ranges from 68% to 98%, and PPV and NPV ranges from 55% to 94% and from 79% to 98%, respectively, using various modalities as the reference gold standard ^26^. When combined with CCTA for corresponding anatomic evaluation, CTA+CTP demonstrates a higher sensitivity and specificity profile than CTA alone ^31– 34^. It is important to consider overall radiation dose when considering CT hemodynamic assessment. CT-FFR requires no additional acquisitions but CTP does. Additional requirements of contrast, along with scanning twice (rest and stress), and drug administration should also be considered. In particular, the effective workflow requires immediate review of the images so that stress images are obtained only when necessary. Weininger *et al*. ^35^ have demonstrated that this is feasible in the ED setting. Nevertheless, for the above-mentioned reasons, implementing stress CTP in the ED setting may be logistically difficult.

There is a significant amount of evidence demonstrating that myocardial infarctions occur not only from severely stenotic vessels but from rupture of “unstable” plaque regardless of the degree of stenosis. It has been shown that plaque features in addition to degree of stenosis are predictors of MACE. But what features make a plaque unstable? Recent work has also centered on identifying plaque characteristics that may deem it “high risk” for rupture. Features such as positive remodeling, low attenuation, and spotty calcification are associated with higher probability of having an acute coronary syndrome ^36^.

Along with recent advances in imaging, there have been advances in the clinical evaluation of patients with chest pain. Improved biomarkers, in the form of high-sensitivity troponins, are under investigation in many centers and may be in widespread use in the coming years. These biomarkers demonstrate a higher sensitivity than conventional troponin assays and have a higher NPV for excluding myocardial infarction within 3 hours ^37, 38^. At least one study ^39^ has shown that length of stay and rates of direct discharge from the ED are equivalent between CCTA and standard of care if high-sensitivity troponins are used in triage.

## Conclusions

CCTA is emerging as a complementary modality in the workup of patients with chest pain. Advances in CT hardware along with software have led to our understanding beyond just anatomical evaluation of stenosis. The ultimate goal of both anatomic and hemodynamic assessment of the coronaries by CT may be widely realized in the not-so-distant future. This will be particularly important in the setting of evaluating patients presenting with acute chest pain in the ED.
